# Effect of Statin Therapy in the Outcome of Bloodstream Infections Due to *Staphylococcus aureus*: A Prospective Cohort Study

**DOI:** 10.1371/journal.pone.0082958

**Published:** 2013-12-23

**Authors:** Luis E. López-Cortés, Juan Gálvez-Acebal, María D. del Toro, Carmen Velasco, Marina de Cueto, Francisco J. Caballero, Miguel A. Muniain, Álvaro Pascual, Jesús Rodríguez-Baño

**Affiliations:** 1 Unidad Clínica de Enfermedades Infecciosas y Microbiología, Hospital Universitario Virgen Macarena, Seville, Spain; 2 Spanish Network for Research in Infectious Diseases, Instituto de Salud Carlos III, Madrid, Spain; 3 Departamento de Microbiología, Universidad de Sevilla, Seville, Spain; 4 Departamento de Medicina, Universidad de Sevilla, Seville, Spain; University Freiburg, Germany

## Abstract

**Introduction:**

Statins have pleiotropic effects that could influence the prevention and outcome of some infectious diseases. There is no information about their specific effect on *Staphylococcus aureus* bacteremia (SAB).

**Methods:**

A prospective cohort study including all SAB diagnosed in patients aged ≥18 years admitted to a 950-bed tertiary hospital from March 2008 to January 2011 was performed. The main outcome variable was 14-day mortality, and the secondary outcome variables were 30-day mortality, persistent bacteremia (PB) and presence of severe sepsis or septic shock at diagnosis of SAB. The effect of statin therapy at the onset of SAB was studied by multivariate logistic regression and Cox regression analysis, including a propensity score for statin therapy.

**Results:**

We included 160 episodes. Thirty-three patients (21.3%) were receiving statins at the onset of SAB. 14-day mortality was 21.3%. After adjustment for age, Charlson index, Pitt score, adequate management, and high risk source, statin therapy had a protective effect on 14-day mortality (adjusted OR = 0.08; 95% CI: 0.01–0.66; p = 0.02), and PB (OR = 0.89; 95% CI: 0.27–1.00; p = 0.05) although the effect was not significant on 30-day mortality (OR = 0.35; 95% CI: 0.10–1.23; p = 0.10) or presentation with severe sepsis or septic shock (adjusted OR = 0.89; CI 95%: 0.27–2.94; p = 0.8). An effect on 30-day mortality could neither be demonstrated on Cox analysis (adjusted HR = 0.5; 95% CI: 0.19–1.29; p = 0.15).

**Conclusions:**

Statin treatment in patients with SAB was associated with lower early mortality and PB. Randomized studies are necessary to identify the role of statins in the treatment of patients with SAB.

## Introduction

Statins act as selective and competitive inhibitors of HMG-CoA reductase. This enzyme converts 3-hydroxy-3-methyl glutaril-coenzime A into mevalonate, which is a precursor of sterols. The hypolipidemic action of statins is due to the inhibition of synthesis of cholesterol in the liver and the increase of the number of liver receptors for LDL cholesterol. In addition to this activity, other so-called pleiotropic effects have been described for statins, including anti-inflammatory, inmunomodulatory, antioxidant and anticoagulant activities [1], [2]. Multiple studies have evaluated the effect of statins in the prevention and prognosis of diverse infectious diseases. Most of these studies ascribed a beneficial effect to exposition to statins [3]–[6]. However, other studies did not found any benefit or even showed harmful effects [7]–[9]. In patients with bloodstream infections (BSI), the use of statins was associated with lower mortality in a recent meta-analysis [10].

*Staphylococcus aureus* is one of the most important etiological agents of both nosocomial and community-onset BSIs [11], [12]; *S. aureus* bacteremia (SAB) is associated with important morbidity and mortality [13]. To our knowledge, there are no studies specifically investigating the effect of statins in the outcome of patients with SAB, in spite of the fact that in vitro and animal model data suggest that they may play a role [14], [15]. It has been hypothesized that this effect could be linked to the inhibition of host cell invasion. This inhibition would facilitate the extracellular activity of antibiotics, avoid intracellular persistence, and so reduce the hematogenous spread of *S. aureus* [14].

The aim of our study is, therefore, to assess the effect of the treatment with statins on the evolution and prognosis of SAB.

## Methods

### Setting, patients, and study design

A prospective cohort study including all episodes of SAB diagnosed in patients aged ≥18 years admitted to Hospital Universitario Virgen Macarena (a 950-bed tertiary hospital located in Seville, Spain) from March 2008 to January 2011 was carried out. A specific “bacteremia program”, carried out by clinical microbiologists and infectious diseases physicians is active in our hospital since 2003; the program includes early personalized reporting of blood culture results and unsolicited management advice for all patients with BSI [16]. Patients with SAB were detected through daily review of microbiology reports. SAB was defined as the isolation of *S. aureus* from at least one blood culture in patients with symptoms or signs of infection. Only one episode (the first) per patient was included in the analysis. Patients were followed for 90 days after the diagnosis of SAB. An experienced team of clinical microbiologists and infectious diseases doctors followed all included patients daily during their admission; the evolution of surviving patients discharged before day 30 was assessed by outpatient clinic visits and/or phone calls. The data were collected by one investigator (LELC) using a structured questionnaire and reviewed by a senior investigator (JRB). This analysis was reported following the STROBE recommendations [17]. The study was approved by the Ethic Committee of Hospital Universitario Virgen Macarena which waived the need to obtained written informed consent from patients because of the observational nature of the study.

### Variables and definitions

The main outcome variable was all-cause 14-day mortality, and the secondary outcome variables were all-cause 30-day mortality, persistent bacteremia (PB; see definition below) and presence of severe sepsis or septic shock at diagnosis of SAB. The outcome variables and their definitions were decided and defined previously. The reasons for choosing 14-day mortality as main outcome variable are explained in the Discussion.

The main exposure variable was therapy with statins. For the purpose of the study, patients were considered to be receiving statins if they had been treated with any drug belonging to this family (including pravastatin, atorvastatin, simvastatin, fluvastatin, lovastatin and pitavastatin) for at least 30 days and were still taking them when SAB was diagnosed. Exposure to statins was assessed by reviewing the charts and by directly interviewing the patients or their relatives. Other exposure variables considered included demographics, types and severity of underlying conditions, type of acquisition of SAB, source of infection, severity of systemic inflammatory response syndrome (SIRS) at presentation, antimicrobial therapy, and support therapy.

We used the Charlson comorbidity index to measure the severity of chronic underlying conditions [18]; this index has been validated as a mortality predictor in SAB [19]. The acute severity of the illness was retrospectively assessed on the day before the diagnosis of SAB using the Pitt bacteremia score, which has also been validated [20]. SAB were considered as hospital-acquired if occurring after 48 hours of hospital stay, and as community-onset in all other cases. The source of infection was established by the agreement between 2 investigators according to clinical and microbiological criteria. Sources of SAB associated with high mortality in previous studies were classified as high-risk sources; these included endocarditis, unknown source, endovascular infections other than catheter-related, central nervous system infections, and respiratory tract infections [21], [22]. The severity of SIRS was classified as sepsis, severe sepsis or septic shock according to standard definitions [23]. Clinical management was considered adequate when it fulfilled all the following criteria: appropriate antimicrobial therapy was administered (at least one active drug was administered during the first 24 hours, and active drugs were administered as definitive therapy); fluid resuscitation was administered in patients with severe sepsis or septic shock according to recommendations [24]; respiratory support was provided according to recommendations [24]; duration of treatment was according to complexity of infection [16], [25], [26]; and the source of infection was removed or drained whenever feasible [16], [27], [28]; otherwise, it was considered non-adequate. Persistent SAB was defined as the isolation of *S. aureus* in blood cultures obtained from peripheral veins for ≥3 days despite appropriate antimicrobial therapy according to susceptibility testing.

### Microbiological Studies

Two or three sets of two blood samples, separated by 20–30 minutes and containing 15 mL of blood each, were drawn in patients who presented fever ≥38°C or when bacteremia was suspected because of clinical signs or symptoms. Blood samples were processed by API Staph system (BioMerieux, NC, USA). The identification of the isolates was assessed by API Staph (BioMerieux, NC, USA), and antimicrobial susceptibility was studied by WIDER (Soria Melguizo, Madrid, Spain) and microdilution according to the recommendations by the Clinical and Laboratory Standards Institute (CLSI) [29].

### Statistical analysis

Crude comparisons were performed by using the Chi squared or Fisher tests as appropriate for percentages, and the Mann-Whitney U test for continuous variables. Multivariate analyses were performed by using logistic regression. Variables with a p value<0.2 in the univariate analysis were included. Selection of variables was performed using a stepwise backward process, variables with a p value<0.2 were kept in the models. Effect modifications between the exposure of interest and other variables were investigated. A propensity score for receiving statins was added to the models. The propensity score (the probability of receiving statins) was calculated using a non-parsimonious multivariate logistic regression model, in which the outcome variable was use of statins; all variables considered as potentially influencing the prescription of statins, including gender, service of admission, type of acquisition, all underlying diseases, recent surgery, and indwelling implants, were included. The validity of the models was assessed by estimating goodness-of-fit to the data with the Hosmer-Lemeshow test, and its discrimination ability with the area under the receiver operating characteristic (ROC) curve. A survival analysis, using time until death (limited to 30 days) or censorship as outcome variable was also performed. Bivariate comparison of Kaplan Meier curves was performed by log rank test, and multivariate analysis was carried out by Cox regression modelling. The software used for the analysis was SPSS v17.0.

## Results

We included 160 episodes of SAB during the thirty five months of the study period. Thirty-three patients (21.3%) were receiving statins at the onset of SAB (all of them had been receiving these drugs for at least one month): 17 (5.15% of those receiving statins) received atorvastatin, 14 (42.4%) simvastatin, and 2 (6%) pravastatin. The epidemiological and clinical features of patients receiving and not receiving statins are shown in Table 1. Diabetes mellitus and catheter-related bacteremia were more frequent among patients who were receiving statins. The treatment and outcomes are also shown in Table 1. Mortality at day 14 and 30 were 21.3% and 28.7%, respectively; crude comparison showed lower 14-day mortality in the statins group; the differences for PB and 30-day mortality were in the limit of significance.

**Table 1:** Features of patients with *Staphylococcus aureus* bacteremia.

Variable	Subcategory	All patients (n = 160)	Statin use (n = 33)	No statin use (n = 127)	p-value
Median age (interquartile range), years		68 (59–77)	67 (63–75)	68 (58–77)	0.4
Male sex		100 (63)	23 (69.7)	77 (60.6)	0.3
Comorbidities					
	Chronic heart failure	28 (17.5)	3 (9.1)	16 (12.6)	0.5
	Chronic pulmonary disease	34 (21.3)	4 (12.1)	24 (18.9)	0.3
	Malignancy	63 (39.4)	6 (18.2)	28 (22)	0.6
	Diabetes mellitus	19 (11.9)	20 (60.6)	43 (33.9)	0.005
	Chronic heart failure	19 (11.9)	3 (9.1)	16 (12.6)	0.5
	Hemodialysis	12 (7.5)	4 (12.1)	8 (6.3)	0.2
	Intravenous drug abuse	5 (3.1)	0 (0)	5 (3.9)	0.2
	Organ transplantation	1 (0.3)	0 (0)	1 (0.8)	0.6
Median Charlson index		2	2	3	0.1
Median Pitt score		2	2	2	0.8
Hospital-acquired infection		93 (58.1)	22 (66.7)	71 (55.9)	0.2
Source of BSI					
	Vascular catheter	69 (43.1)	23 (69.7)	46 (36.2)	0.001
	Unknown source	30 (18.8)	4 (12.1)	26 (20.5)	0.2
	Respiratory tract	11 (6.9)	0 (0)	11 (8.7)	0.08
	Skin and/or soft tissue	24 (15)	6 (18.2)	18 (14.2)	0.5
	Endocarditis	4 (2.5)	0 (0)	4 (3.1)	0.9
	Others	22 (13.7)	0 (0)	22 (100)	0.1
	High risk source#	48 (42.8)	4 (12.1)	44 (34.6)	0.01
Methicillin-resistant *S. aureus*		26 (16.3)	4 (12.1)	22 (17.3)	0.4
ICU admission		42 (26.3)	10 (30.3)	32 (25.2)	0.5
Adequate management		145 (90.6)	30 (90.9)	115 (90.6)	0.9
Outcome variables					
	14-day mortality	34 (21.3)	2 (6)	32 (25.2)	0.01
	30-day mortality	46 (28.7)	5 (15.2)	42 (32.3)	0.05
	Persistent bacteremia*	34/150 (22.7)	3/31 (9.7)	31/119 (26.1)	0.05
	Severe sepsis or septic shock	38 (23.7)	6 (18.2)	33 (25.2)	0.5

* Patients who died within 96 hours are excluded.

# High risk source: endocarditis, unknown source, endovascular infections other than catheter-related, central nervous system infections, and respiratory tract infections.

Data are expressed as number of cases (percentage) except where specified.

The univariate analysis of the association of exposure to categorical variables and 14-day mortality is shown in Table 2. Apart from statin use, other variables showing a crude association with 14-day mortality were the source of bacteremia, non-adequate clinical management, and presentation with severe sepsis and septic shock. As regards the continuous variables [measured as median value (interquartile range)], age and Pitt score were significantly higher among patients who died [age in years: 75 (63–83) vs. 66 (58–75), p = 0.01; Pitt score: 3 (2–4) vs. 1 (0–2), p<0.001, respectively], but Charlson index was not [2 (2–4) vs. 2 (1–3), p = 0.09].

**Table 2:** Univariate analysis of 14-day mortality among patients with *S. aureus* bacteremia according to exposure to different categorical variables.

Variable	Subcategory	No. dead/No. exposed(percentage)	RR (95% CI)	P value
Gender	Male	19/100 (19)	Ref.	
	Female	15/60 (25)	0.76 (0.41–1.38)	0.3
Source				
	Catheter	6/69 (8.7)	Ref.	Ref.
	Respiratory	8/11 (72.7)	8.36 (3.58–19.48)	<0.001
	Unknown	9/30 (30)	3.45 (1.34–8.83)	0.009
	Skin and/or soft tissue	6/24 (25)	2.87 (1.02–8.06)	0.04
	Endocarditis	2/4 (50)	5.75 (1.65–19.92)	0.01
	Others	2/12 (16.7)	1.91 (0.43–8.44)	0.86
Type of acquisition				
	Community-onset	14/67 (20.9)	Ref.	
	Nosocomial	20/93 (21.5)	0.97 (0.53–1.78)	0.9
Persistent bacteremia¶				
	Yes	7/34 (20.6)	1.41 (0.53–3.72)	0.4
	No	18/116 (15.5)	Ref.	
Severe sepsis or septic shock				
	Yes	21/35 (60)	12.92 (5.32–31.38)	<0.001
	No	13/125 (10.4)	Ref.	
Susceptibility				
	MRSA	5/26 (19.1)	Ref.	
	MSSA	29/134 (21.6)	1.12 (0.48–2.63)	0.7
Empirical treatment				
	Appropriate	24/122 (19.7)	Ref.	
	Non-appropriate	10/38 (26.3)	1.33 (0.70–2.54)	0.3
Clinical management				
	Adequate	25/145 (17.2)	0.24 (0.06–0.94)	<0.001
	Non-adequate	9/15 (60)	Ref.	
Statins use				
	Yes	2/33 (6.1)	0.24 (0.06–0.94)	0.01
	No	32/127 (25.2)	Ref.	

¶ Considered only among surviving patients at 96 hours.

MRSA: Methicillin-resistant *S. aureus*. MSSA: Methicillin-susceptible *S. aureus*.

**Figure 1:**
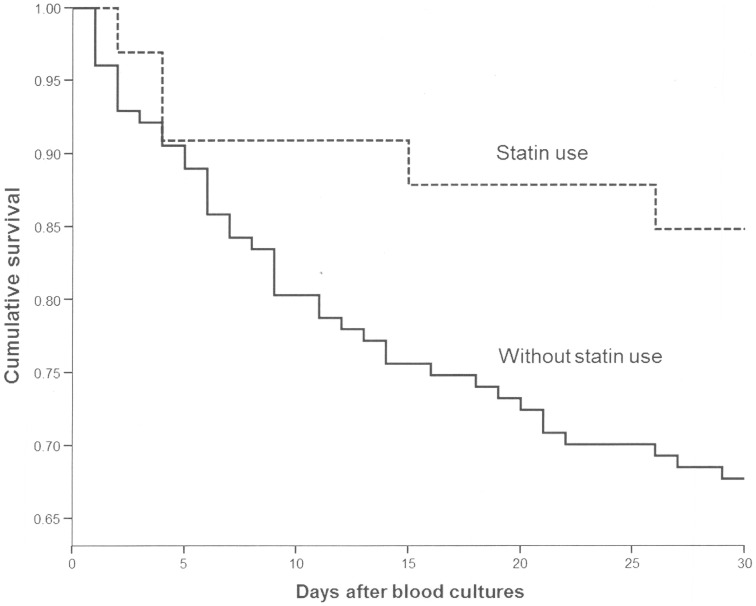
Kaplan-Meier survival curves for patients receiving and not receiving statins.

We calculated a propensity score for receiving statins. The model showed a p value of 0.42 for the Hosmer-Lemeshow goodness-of-fit test, and an area under the ROC curve of 0.877, showing good predictive ability for receiving statins. In order to control for confounding in the association between statins and 14-day mortality, we performed several multivariate analysis by logistic regression. The variables introduced were age, Charlson index, Pitt score, high risk source, adequate clinical management, and statin use, plus the propensity score. Neither presentation with severe sepsis/septic shock nor PB was introduced in the model because these variables are in the pathogenic pathway between development of SAB and death, and exposure to statins preceded them. Because the source of bacteremia was an important predictor of outcome (Table 2) and the distributions of SAB sources were different among patient exposed and not exposed to statins, we performed different analysis to control for this confounder. To do so, we performed 4 multivariate models for which the variable source was defined in different ways (Table 3). Statin therapy was independently associated with statin therapy in all of them. All models showed a high prediction ability; as an example, model 1, in which source was classified as high or low-risk, showed a p value of 0.63 for the Hosmer-Lemeshow goodness-of-fit test, and an area under the ROC curve of 0.90. Additionally, we performed another model (not shown) in which source was defined as a polichotomous variable (including catheter, which was taken as reference, respiratory tract, unknown, and others); in this model, statin therapy remained associated with lower 14-day mortality (adjusted OR = 0.08; CI 95%: 0.01–0.83; p = 0.03). Finally, because the models might be overfitted due to the high number of variables introduced in relation to the number of patients with the event, we also performed several models in which the variables were added to statins in a forward approach to a limit of 3. Statin therapy showed a protective effect for 14-day mortality in all of them (data not shown). We also performed a survival analysis until day 30. The Kaplan Meier curves are shown in figure 1; the log rank value for mortality among patients who received and not received statins was 0.06. The Cox regression model including the propensity score, did not show association of statin use with 30-day mortality (adjusted HR = 0.5; 95% CI: 0.19–1.29; p = 0.15).

**Table 3:** Different multivariate models for 14-day mortality.

	Model1		Model 2		Model 3		Model 4	
	OR (95% CI)	P value	OR (95% CI)	P value	OR (95% CI)	P value	OR (95% CI)	P value
Statins use	0.08 (0.01–0.67)	0.02	0.09 (0.01–0.70)	0.02	0.08 (0.01–0.74)	0.03	0.07 (0.01–0.49)	0.008
Age (per unit)	1.05 (1.01–1.09)	0.007	1.05 (1.01–1.08)	0.02	1.06 (1.02–1.10)	0.006	1.05 (1.01–1.09)	0.01
Charlson index (per unit)	1.26 (0.98–1.62)	0.06	1.20 (0.93–1.55)	0.16	1.20 (0.93–1.53)	0.16	1.21 (0.96–1.55)	0.11
Pitt score (per unit)	1.79 (1.31–2.46)	<0.001	1.82 (1.34–2.46)	<0.001	1.81 (1.33–2.49)	<0.001	1.77 (1.34–2.41)	<0.001
Source according to below definition*	5.72 (2.07–15.79)	0.001	12.86 (2.54–65.21)	0.002	0.13 (0.04–0.46)	0.002	1.91 (0.63–5.79)	0.26
Adequate management	0.09 (0.02–0.42)	0.002	0.09 (0.02–0.38)	0.001	0.06 (0.01–0.29)	0.001	0.12 (0.03–0.46)	0.002

* Source was defined as a dichotomous variable as follows. Model 1: high risk source (endocarditis, unknown source, endovascular infections other than catheter-related, central nervous system infections, and respiratory tract infections) vs. low risk source. Model 2: respiratory source vs. other sources. Model 3: catheter-related source vs. other sources. Model 4: Unknown source vs. known source.

The propensity score for including statins was included in all of them. The definition for the variable source was different in each model (see footnote).

We also performed multivariate analyses for secondary outcome variables. An independent relationship between statin use and these variables could not be demonstrated using a similar strategy, neither for 30-day mortality (adjusted OR = 0.35; 95% CI: 0.10–1.22; p = 0.10) nor for presentation with severe sepsis or septic shock (adjusted OR = 0.89; CI 95%: 0.27–2.94; p = 0.8). However, statins use showed a protective effect for PB (adjusted OR = 0.89; 95% CI: 0.27–1.00; p = 0.05).

In the subgroup analyses, statin therapy showed the following association to 14-day mortality: among nosocomial SAB, OR = 0.08; CI 95%: 0.005–1.27; among community-onset SAB, OR = 0.81; CI 95%: 0.21–3.27; among patients aged ≤60 years, OR = 0.43; CI 95%: 0.02–6.95; among those older than 60 years, OR = 0.25; CI 95%: 0.006–0.98; among patients with a Charlson index ≤1, OR = 0.38; CI 95%: 0.02–6.23; finally, among those with a Charlson index ≥2, OR = 0.25; CI 95%: 0.06–0.98.

## Discussion

We found an association between statin therapy and reduced risk of early death among patients with SAB. Such association could not be demonstrated for 30-day mortality.

We chose 14-day mortality as our main outcome variable because our hypothesis was that statin therapy may have an impact in early, acute infection-related mortality, and we considered this time frame would adequately represent the biological plausible window of effect, as has been recommended [30]. Since mortality in patients with SAB may be due either to a direct effect of the infection (because of septic shock, respiratory distress, disseminated intravascular coagulation) or to later complications related to endocarditis or metastatic lesions, and also to decompensation of underlying conditions or other non-infection-related caused, early mortality is more probably related to the direct impact of the infection rather than to late metastatic complications or unrelated issues.

Patients receiving statins were similar to those not exposed to these drugs in demographic features and underlying conditions except diabetes mellitus. This was expected because diabetes mellitus is frequently associated with dyslipidemia, which would increase the probability of receiving statins due to a higher cardiovascular risk. Patients with statins had a higher frequency of catheter-related SAB and a trend towards lower frequency of respiratory tract infection-related SAB, which would clearly reduce their baseline risk of mortality, since catheter-related BSI is usually associated with lower mortality while the opposite is seen with respiratory tract infections [13]. These and other potential confounders were controlled by performing multivariate analyses in which a propensity score for receiving statins was also used.

The association of statin treatment with lower mortality in different types of infection has been previously described, but there are scarce data about *S. aureus*. A recent meta-analysis assessed the effect of statins on all-cause mortality among patients with severe infections [10]; the pooled analysis showed an overall survival benefit in patients receiving statins. Bacteremia-related mortality was reported in 4 of these studies; a meta-analysis of this subgroup of studies also showed a lower rate of bacteremia-related mortality in patients receiving statins (OR = 0.33; 95% CI: 0.09–0.75); specific data for SAB were not provided. In a cohort of 388 patients with BSI of diverse etiologies, statins use was associated with a 17% decrease in the attributable mortality [31]; however, in the sub-analysis performed in SAB patients the decrease in mortality was not statistically significant (no multivariate analysis in patients with SAB was performed). To our knowledge, there are no other studies specifically addressing the impact of statins on mortality among patients with SAB, although there are some features of SAB that worth a specific investigation: SAB is more frequently persistent and complicated than bacteremia due to other microorganisms [32]–[34], and clinical management has been consistently shown to influence the outcome [23]–[26], [35].

The pathophysiological basis for the potential association between statin treatment and lower mortality in some infectious diseases is still unknown. In our study, patients under statin therapy developed severe sepsis and septic shock with less frequency, but the difference was not statistically significant, and multivariate analysis could not show a protective effect of statins for developing severe systemic inflammatory response syndrome (SIRS). This is in contrast to previous observations in patients with infections due to other microorganisms [36], [37]. Beyond the potential effect on the development of severe SIRS, we found that patients receiving statins had a lower frequency of PB. PB has been associated with increased mortality in previous studies of SAB [30], [38], [39]. PB has been shown to be related to some hosts' features as well as to clinical management [39], [40]. However, the molecular mechanisms involved in PB are poorly known. It is known that secretion of platelet microbicidal proteins (PMPs) and intracellular killing by neutrophil-associated oxidative and nonoxidative mechanisms are involved in the initial phases of vascular invasion leading to bacteremia [27], [41], [42]. On the other hand, Xiong et al. [34] showed that MRSA causing PB tend to be more resistant to key innate cationic host defense molecules from both neutrophils (e.g., hNP-1) and platelets (e.g., tPMP-1), have more enhanced membrane fluidity, and substantially greater adhesion to fibronectin, fibrinogen, and endothelial cells. Also, isolates causing PB adhere better to host cells (i.e., endothelium) and matrix ligands relevant to endovascular pathogenesis (i.e., fibrinogen and fibronectin) [34]. Simvastatin has been shown to inhibit cell invasion by *S. aureus* in vitro [14] and to interfere with exotoxine-induced leukocyte-endothelial cell interactions [15]. Whether the lower rate of PB in patients treated with statins, if confirmed, might be related to any of these effects or others [43], would merit being investigated.

Our study must be interpreted considering its limitations. Since it is not a randomized study, confounding due to unmeasured variables may not have been controlled. However, we made a great effort to control the potential confounding factors, including a propensity analysis and performing several multivariate models. The healthy user effect (patients receiving statins may be subject to better medical follow-up and a healthier lifestyle) needs to be taken into account [44]. Although our propensity score analysis could not discard this effect, we do not think it is a relevant issue since the effect was also significant in patients with nosocomial infections. Our study does not allow determining whether the effect of statins is immediate or it requires a minimum treatment time to be established, as it is the case of the hypolipidemic effect. Finally, since most of the patients were treated with atorvastatin or simvastatin (91%), we cannot assess differences in the impact of specific statins. The fact that we could not find an effect on 30-day mortality may be due to a lack of longer effect of statins, or to the limited statistical power of the study. Finally, we did not study the molecular features of the *S. aureus* isolates.

## Conclusion

In conclusion, in this prospective observational study, treatment with statins showed a protective effect for 14-day mortality and PB among patients with BSA. Further studies are needed to determine the role of statins in the treatment of patients with BSA.
